# IFN-λ1 with Th17 axis cytokines and IFN-α define different subsets in systemic lupus erythematosus (SLE)

**DOI:** 10.1186/s13075-017-1344-7

**Published:** 2017-06-15

**Authors:** Vilija Oke, Susanna Brauner, Anders Larsson, Johanna Gustafsson, Agneta Zickert, Iva Gunnarsson, Elisabet Svenungsson

**Affiliations:** 1 Rheumatology Unit, Department of Medicine, Karolinska Institutet, Karolinska University Hospital, SE-171 76 Stockholm, Sweden; 2 Clinical Chemistry research group, Department of Medical Sciences, Uppsala University, Uppsala, Sweden

**Keywords:** Systemic lupus erythematosus, Interferon-α, Interferon-λ1, Interleukin-17, Interleukin-23, IP-10

## Abstract

**Background:**

Interferon (IFN)-α is thought to have a pivotal role in systemic lupus erythematosus (SLE), and type III IFNs (IFN-λ) were recently also associated with SLE. In this study, we measured levels of IFN-α, IFN-λ1, and related cytokines, such as IL-17A, IL-23, and interferon-γ-induced protein 10 (IP-10), in a Karolinska University Hospital cohort of patients with SLE and control subjects. The objective of the study was to investigate if cytokine measurements could identify different subsets of patients with active SLE and higher disease damage.

**Methods:**

We included 261 patients with SLE and 261 population control subjects. All participants underwent a standardized clinical examination. Medical files were reviewed. Patients with SLE were assessed for current organ manifestations, disease activity, and damage. Routine blood parameters, complement levels, and serology were analyzed at the time of inclusion. Levels of IFN-λ1, IFN-α, IL-17A, IL-23, and IP-10 were measured by enzyme-linked immunosorbent assay.

**Results:**

IFN-λ1 and IFN-α were detected in 29% and 44% of patients, respectively, but their levels did not correlate. High serum levels of IFN-λ1 were positively associated with antinucleosome antibodies and lymphopenia but negatively with musculoskeletal damage. Positive correlations between levels of IFN-λ1, IL-17A, and IL-23 were observed. Patients with high levels of these three cytokines had more disease damage, especially renal impairment. High levels of IFN-α were associated with mucocutaneous disease; leukopenia; and low complement, Ro/SSA, and La/SSB. Vascular events and antiphospholipid antibodies were uncommon. We identified two subgroups with high disease activity: one with double-high IFN-λ1 and IFN-α and another with IP-10^high^. The former had more neuropsychiatric manifestations, and the latter had more arthritis. Increased levels of both types I and III IFNs were found in a proportion of population control subjects. Therefore, high IFN levels do not seem to be SLE-specific biomarkers.

**Conclusions:**

Measurements of circulating IFN-λ1 and IFN-α define subsets of patients with SLE with different characteristics. Levels of IFN-λ1 correlate with T-helper type 17 cytokines and identify a subgroup with more damage. High disease activity is associated with either simultaneous upregulation of IFN-λ1 and IFN-α or independently with IP-10. Our findings could be of major importance when tailoring therapy for patients with SLE with agents targeting IFN pathways.

## Background

Systemic lupus erythematosus (SLE) is a heterogeneous autoimmune disease. One hallmark of SLE is the presence of autoantibodies against nuclear constituents (antinuclear antibodies [ANA]), including anti-double-stranded DNA (anti-dsDNA). Organ involvement is diverse, but joints, skin, mucosa, kidneys, and the nervous system are commonly involved. Typical laboratory aberrations include cytopenias and complement activation. SLE diagnosis overlaps with other autoimmune conditions in subsets of patients, such as antiphospholipid syndrome (APS) or Sjögren’s syndrome. Predictors of specific organ involvement or damage are still insufficiently identified [1–3].

Type I interferons (IFNs; particularly IFN-α) play a major role in SLE pathogenesis, and type II IFNs are also of importance [4, 5]. A proportion of patients displays increased serum levels of IFN-α or an upregulation of the IFN-regulated genes (IFN signature) [6–8]. There are 13 known subtypes of IFN-α, and this fact constitutes one of multiple technical challenges in detection of IFN-α subtypes. Thus, many researchers rely on indirect measurements of the IFN signature [9]. Interestingly, the gene signatures of type III (IFN-λ) and type I IFNs overlap [10]. Increased levels of IFN-λ have been reported in SLE [11]. Therefore, both these IFN subsets are of interest in the context of SLE and the IFN signature.

Four molecules belong to the IFN type III (IFN-λ) group: IFN-λ1, IFN-λ2, IFN-λ3 (also referred to as IL-29, IL-28a, and IL-28b, respectively), and IFN-λ4 [12, 13]. IFN-λ is typically produced by virus-infected epithelial cells or plasmacytoid dendritic cells, but other antigen-presenting cells, T-helper type 17 (Th17) cells, keratinocytes, and neutrophils are additional sources of IFN-λ [14–16]. There is a single IFN-λ receptor, which is expressed mainly on cells of epithelial origin, such as skin, gut, kidney epithelium, and neutrophils [12, 17].

Th17 cells are important in SLE, and we previously reported that high levels of Th17 cytokines (interleukin [IL]-17 and IL-23) are associated with poor renal prognosis [18]. Also, Th17 cells have been reported to produce IFN-λ1 in psoriatic lesions [15]. However, the relationship between Th17 and IFN-λ has not been explored in SLE.

Chemokine C-X-C motif chemokine 10 (CXCL10)/interferon-γ-induced protein 10 (IP-10), initially described as an IFN-γ-inducible protein, is often considered an indirect biomarker of type I IFNs. IP-10 is upregulated in SLE and is associated with disease activity and specific clinical manifestations [9, 19, 20].

SLE is a heterogeneous disease. Approximately 40% of patients develop nephritis, 80% have arthritis and/or mucocutaneous manifestations, and 70% demonstrate hematological abnormalities [1, 2]. Many investigators have hypothesized that different pathogenetic mechanisms and molecules may drive SLE subgroups and result in the observed clinical and serological diversity. Moreover, on the basis of early data derived from trials on IFN-α therapies, researchers have reported that only a subgroup of patients with SLE with the IFN signature respond to the therapy [21].

The aim of this study was to investigate if type I and type III IFNs drive SLE independently or in parallel, as well as which clinical parameters are associated with upregulation of these cytokines. In addition, we explored if there are intercorrelations between the IFNs, including IP-10, and the IL-17/IL-23 system.

In this article, we present our findings on the levels and clinical associations of IFN-λ1 and IFN-α in a large cohort of patients with SLE and a matched population of control subjects. Additionally, we describe the relationship between the IFN subtypes and IL-17A, IL-23, and IP-10 in regard to clinical and laboratory parameters of SLE.

## Methods

### Study population

This cross-sectional study included 261 consecutive patients with SLE from the Karolinska SLE cohort. All patients fulfilled at least four of the 1982 revised American College of Rheumatology classification criteria for SLE [22]. Patients were ≥18 years old, and no other exclusion criteria were applied. We identified 261 control subjects in the population registry who were matched for age, sex, and geographical region to the patients with SLE. Among control subjects, a diagnosis of SLE was the only exclusion criterion. All participants underwent a structured investigation by a rheumatologist. Clinical and routine laboratory data were documented at the time of inclusion. SLE disease activity was assessed by using both the Systemic Lupus Erythematosus Disease Activity Index (SLEDAI) and the Systemic Lupus Activity Measure (SLAM). The latter captures more subjective symptoms such as fatigue and musculoskeletal pain [23]. Organ damage was assessed with the Systemic Lupus International Collaborating Clinics/American College of Rheumatology Damage Index (SDI) [24–26]. Definitions of the specific organ manifestations were recorded according to the SLAM and SLEDAI instruments, with slight modifications of some items, as defined below [26, 27]. Patients with SLAM or SLEDAI scores >6 were considered to have active disease. Mucocutaneous activity was defined as a positive score for any of SLAM items 4–7. Damage was assessed using SDI definitions, with the exception of renal damage, which was defined as a glomerular filtration rate (GFR) ≤60 ml/minute/1.73 m^2^, according to the Modified Diet in Renal Disease formula, or terminal renal failure due to nephritis (on dialysis or with a transplant) [28, 29]. Venous thromboembolism (VTE) included both pulmonary embolism and deep vein thrombosis. Our definition of any vascular event (VE) included any objectively verified arterial or venous event, including stroke, transitory ischemic attack, myocardial infarction, angina, peripheral vascular ischemia, or VTE, as previously specified [30]. Serum samples were collected at inclusion after overnight fasting, aliquoted, and stored at −70 °C until analysis.

### Laboratory methods

All blood and urine chemistry analysis was performed according to standard routine at the time of inclusion at the internationally certified Karolinska University Hospital laboratory. ANA were analyzed by indirect immunofluorescence on HEp-2 cells (Immuno Concepts, Sacramento, CA, USA). Antibodies to specific nuclear antigens (anti-dsDNA, antinucleosomes, anti-Ro52/SSA, anti-Ro60/SSA, anti-La/SSB, anti-Smith) and phospholipids (anticardiolipin antibodies immunoglobulin (aCL IgG), and anti-β_2_-antiglycoprotein domain 1 antibodies IgG [aβ_2_GP1IgG]) were analyzed by multiplex bead technology (Luminex, Austin, TX, USA) using the BioPlex 2200 system (Bio-Rad Laboratories, Hercules, CA, USA). The cutoff for aCL and aβ_2_GP1 fulfilled the 99th percentile as described previously [31]. Lupus anticoagulant (LA) was determined by the modified dilute Russell’s viper venom time method (Biopool, Umeå, Sweden) using Bioclot LA (Trinity Biotech, Co.Wicklow, Ireland). aCL, aβ_2_GP1, and LA are together referred to as antiphospholipid antibodies (aPL).

### Detection of cytokines

IFN-λ1 and IFN-α levels in sera were measured by enzyme-linked immunosorbent assay (ELISA) according to the manufacturer’s instructions. For the IFN-λ1 assay, a mouse anti-IFN-λ1 IgG2A capture monoclonal antibody (MAB15981; R&D Systems, Minneapolis, MN, USA) and affinity-purified goat polyclonal IgG (BAF1598; R&D Systems) were used for coating and detection, respectively. The reagents had up to 100% cross-reactivity with IFN-λ3. IFN-α subtypes were measured using a pan-IFN-α ELISA detection kit. The detected IFN-α subtypes included 1, 2, 4, 5, 6, 7, 8, 10, 13, 14, 16, and 17 (3425-1A-20; Mabtech AB, Nacka Strand, Sweden), representing all but one IFN-α (IFN-α21). Therefore, we refer to our findings hereinafter as IFN-α.

In short, high-binding 96-well Nunc plates (Thermo Fisher Scientific, Waltham, MA, USA) were coated with capture antibody at 8 μg/ml for IFN-λ1 and 4 μg/ml for IFN-α in a carbonate buffer, pH 9.6, and incubated at +4 °C overnight. Plates were blocked (5% milk powder, 0.05% Tween in PBS) for 1 h, then washed and incubated overnight at +4 °C with patient sera, diluted 4:1 for detection of IFN-λ1 and 2:1 for IFN-α in a dilution buffer (3652-D2; Mabtech AB). Recombinant human IFN-λ1 (1598-IL; R&D Systems) and IFN-α included in the kit were used for derivation of standard curves. Ten serial dilutions were performed. Wells containing only buffer were used to determine the level of background. Negative control samples from five healthy control subjects were used when setting up and titrating the ELISA. Samples were run in duplicates. Biotinylated detection antibodies were added at a concentration of 0.4 μg/ml for IFN-λ1 and 1 μg/ml for IFN-α and incubated for 1 h in room temperature (RT). After a washing step, streptavidin-alkaline phosphatase diluted at 1:1000 was added and incubated for 1 h at RT. Afterward, a substrate solution (N1891, SIGMAFAST Protease Inhibitor Cocktail; Sigma-Aldrich, St. Louis, MO, USA) was added, and optical density was measured after 6 h and overnight at 405 nm.

Human IL-17A, IL-23, and IP-10 were analyzed with commercial sandwich ELISAs (DY317, DY1290, and DY266; R&D Systems) according to the manufacturer’s instructions. Streptavidin-HRP followed by addition of substrate (P3804 *o*-phenylenediamine dihydrochloride; Sigma-Aldrich) was used as a detection reagent, and optical density was measured at 450 nm.

The titration allowed us to detect IFN-α levels down to 2 pg/ml and 16 pg/ml for IFN-λ1. However, accurate detection levels recommended by the manufacturer and also in relation to the estimated standard curves of the ELISA were set to the following: 36 pg/ml for IFN-α, 300 pg/ml for IFN-λ1, 10 pg/ml for IL-17, 100 pg/ml for IL-23, and 18 pg/ml for IP-10. Values below the cutoff were denoted 50% of the cutoff value and transformed logarithmically with base 10 (log_10_).

### Statistics

Student’s *t* test was used to compare normally distributed continuous variables, and the Mann-Whitney *U* test or the Wilcoxon/Kruskal-Wallis tests were used for non-normally distributed and nonparametric variables. For comparison between proportions, we used Pearson’s chi-square test or a two-tailed Fisher’s exact test. Correlations were calculated by Spearman’s rank correlation analysis. *p* Values <0.05 were considered significant. JMP software (SAS Institute, Cary, NC, USA) was used for all statistical analyses.

## Results

### Basic characteristics

The study included 261 patients with SLE and 261 population control subjects. Almost half (49%) of the patients had high disease activity according to the SLAM and 26% according to the SLEDAI. At inclusion, disease damage was present in 64% of patients. Clinical characteristics are presented in Table 1.

**Table 1:** Characteristics of the study subjects

Characteristics	Patients with SLE (*n* = 261)	Control subjects (*n* = 261)	*p* Value
Age, years, mean (SD)	47.6 (14.8)	47.8 (14.7)	ns
Sex, male/female	19/242	14/262	ns
Smoker, %	18.5	14	ns
Features of SLE, ever, %			
Positive ANA	99	nd	nd
Positive anti-dsDNA	36	2	<0.0001
Malar rash	52		
Discoid rash	20		
Photosensitivity	69		
Oral ulceration	34		
Arthritis	84		
Pleuritis	34		
Pericarditis	17.5		
Nephritis	42		
Leukopenia	49		
Lymphopenia	50		
Thrombocytopenia	20		
Hemolytic anemia	5		
Neuropsychiatric	11.5		
SLAM >6	49		
SLEDAI >6	26		
SDI >0	64		
Individuals with detectable cytokines, %			
IFN-α	44	33	0.008
IFN-λ1	29	20	0.01
IFN-α and IFN-λ1	14.5	7.5	0.01
IL-17A	12	5	0.006
IL-23	58	41	0.0001
IP-10	100	100	ns

*Abbreviations: ANA* Antinuclear antibodies, *dsDNA* Double-stranded DNA, *IFN* Interferon, *IL* Interleukin, *IP-10* Interferon-γ-induced protein 10, *nd* Not done, *ns* Nonsignificant, *SDI* Systemic Lupus International Collaborating Clinics/American College of Rheumatology Damage Index, *SLAM* Systemic Lupus Activity Measure, *SLE* Systemic lupus erythematosus, *SLEDAI* Systemic Lupus Erythematosus Disease Activity Index

Statistical analysis was performed by Student’s *t* test, nonparametric Wilcoxon/Kruskal-Wallis test, and Pearson’s/Fisher’s exact tests

### Serum levels of IFN-λ1, IFN-α, IL-23, IL-17A, and IP-10

**Fig. 1:**
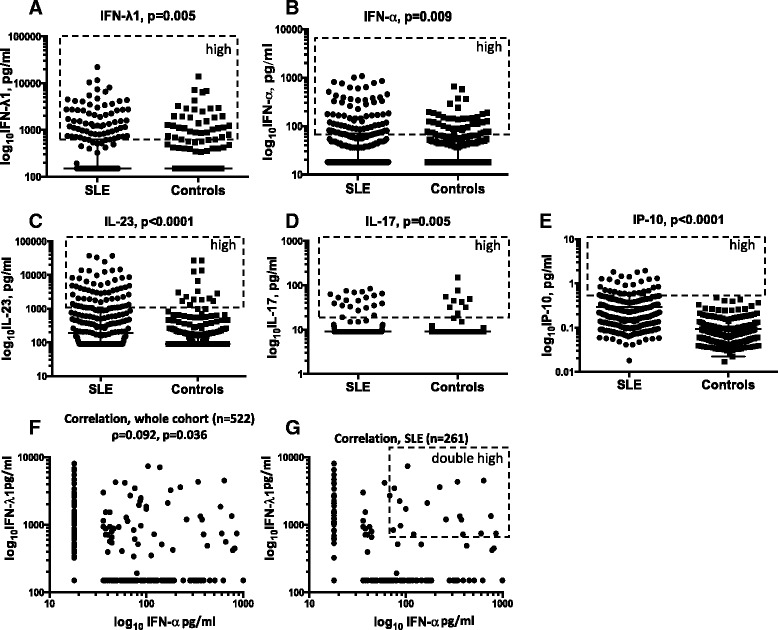
Levels of cytokines in patients with systemic lupus erythematosus (SLE) and a population of control subjects. Levels of (**a**) interferon (IFN)-λ1 and (**b**) IFN-α are significantly higher in patients with SLE (Mann-Whitney *U* test). **c**–**e** The cytokines interleukin (IL)-17A, IL-23, and interferon-γ-induced protein 10 (IP-10) are all expressed at higher levels in patients with SLE (Mann-Whitney *U* test). **f** The levels of IFN-λ1 are independent of the levels of IFN-α in the analysis of the whole cohort (*n* = 522) (Spearman’s rank correlation test). **g** No correlation was observed between the levels of IFN-λ1 and IFN-α in patients with SLE (Spearman’s rank correlation test). Data on cytokine concentrations are presented in log_10_ scale. The *dashed boxes* indicate individuals with high expression levels of each investigated cytokine (**a**–**e**) or double-high IFN-λ1 and IFN-α (**g**). The limit for high expression was set at the 75th percentile for IFN-λ1, IFN-α, IL-23, and IP-10. For IL-17A, the limit for high was set at the 90th percentile. Medians are indicated by *horizontal lines*, and IQRs are indicated by *whiskers* (90th percentile for IL-17A)

IFN-λ1 was detected in 29% of patients and 20% of control subjects, whereas IFN-α was detected in 44% and 33%, respectively. In only 14.5% of patients and 7.5% of control subjects, both IFN-λ1 and IFN-α were detected concomitantly. In substantial proportions of both patients and control subjects (41% and 55%, respectively), neither IFN-λ1 nor IFN-α was expressed at measurable levels. IL-17A was detected in 12% and 5% and IL-23 in 58% and 41% of patients and control subjects, respectively, whereas IP-10 could be measured in all investigated individuals. The levels of all cytokines were generally higher among patients with SLE than in control subjects (Table 1 and Fig. 1a–e).

### Correlations between cytokine levels

We investigated intercytokine correlations. There was no correlation between the levels of IFN-λ1 and IFN-α in the cohort (*n* = 522, ρ = 0.09, *p* = 0.03) (Fig. 1f). No correlation was observed when patients and control subjects were examined separately (Fig. 1 g and data not shown). Both in the whole cohort and separately in patients with SLE, the levels of IFN-λ1 correlated with IL-17A and IL-23, and concentrations of the latter two correlated with each other (ρ = 0.41, ρ = 0.44, ρ = 0.38, *p* < 0.0001 for the whole cohort). Values for SLE subsets are provided in Table 2. Weak correlations were observed among IFN-λ1, IL-17A, and IL-23 in control subjects (ρ = 0.2, *p* ≤ 0.001 for all; data not shown). The IP-10 levels weakly correlated with IFN-λ1 (ρ = 0.20), IL-23 (ρ = 0.32), and IL-17A (ρ = 0.20, *p* < 0.0001 for all). Also, the levels of IP-10 correlated weekly with disease activity as measured by SLEDAI (Table 2).

**Table 2:** Correlations between the levels of investigated cytokines in patients with systemic lupus erythematosus

Cytokine	IFN-λ1	IL-23	IL-17A	IP-10	IFN-α
IFN-λ1	–	ρ = 0.57 *p* < 0.0001	ρ = 0.50 *p* < 0.0001	ρ = 0.175 *p* = 0.005	ns
IL-23	ρ = 0.57 *p* < 0.0001	–	ρ = 0.47 *p* < 0.0001	ρ = 0.21 *p* = 0.0007	ρ = 0.16 *p* = 0.01
IL-17A	ρ = 0.51 *p* < 0.0001	ρ = 0.47 *p* < 0.0001	–	ρ = 0.2 *p* = 0.0007	ns
IP-10	ρ = 0.175 *p* = 0.005	ρ = 0.21 *p* = 0.0007	ρ = 0.2 *p* = 0.0007	–	ns
SLAM	ns	ns	ns	ρ = 0.16 *p* = 0.01	ns
SLEDAI	ns	ns	ρ = -0.13 *p* = 0.037	ρ = 0.21 *p* = 0.0007	ns
SDI	ns	ns	ns	ρ = 0.13 *p* = 0.047	ρ = −0.14 *p* = 0.028

*Abbreviations: IFN* Interferon, *IL* Interleukin, *IP-10* Interferon-γ-induced protein 10, *ns* Nonsignificant, *SDI* Systemic Lupus International Collaborating Clinics/American College of Rheumatology Damage Index, *SLAM* Systemic Lupus Activity Measure, *SLEDAI* Systemic Lupus Erythematosus Disease Activity Index

Statistical analysis was performed by Spearman’s rank correlation test

### High cytokine levels identify patients with different phenotypes

We investigated characteristics of the subgroups of patients with detectable cytokine levels and observed some trends. Therefore, we hypothesized that individuals with high levels of cytokines, indicative of higher inflammatory activity, might have distinct characteristics.

We identified patients with high levels (i.e., ≥75% or third quartile) of each investigated cytokine, and in further comparisons we grouped the patients accordingly. The limit for “high expression” was thus 628 pg/ml for IFN-λ1, 71 pg/ml for IFN-α, 335 pg/ml for IP-10, and 966 pg/ml for IL-23. For IL-17A, we set the top 90th percentile as a limit for “high,” which was 13 pg/ml.

There were 19 individuals (7%) who had high levels of both IFN-λ1 and IFN-α. Levels of IFN-λ1 correlated to IL-17A and IL-23, and there were 20 “triple-high” patients (7.5%). Consequently, we hypothesized that these groups could have a certain SLE profile and investigated them accordingly.

The following patient subgroups were identified and analyzed separately: IFN-λ1^high^; IFN-α^high^; double-high IFN-λ and IFN-α; IP-10^high^; and triple-high IFN-λ1, IL-17A, and IL-23. In the following statistical analysis, each subgroup was compared with the rest of the cohort. Analysis was performed for all SLAM, SLEDAI, and SDI items, as well as routine blood chemistry and autoantibodies. Only statistically significant results are provided in the figures and tables and discussed in the text.

#### Features associated with high levels of IFN-λ1

Patients with high levels of IFN-λ1 (*n* = 65) were compared as a group with the rest of the SLE cohort (*n* = 196) (Table 3). We found that there were fewer smokers in the IFN-λ1^high^ group. The IFN-λ1^high^ patients were more often positive for antinucleosome antibodies. They had a similar frequency of objective arthritis, but significantly fewer patients had musculoskeletal damage. A lower lymphocyte count was a distinct laboratory characteristic of this group. Furthermore, IFN-λ1^high^ patients had higher levels of IL-17A, IL-23, and IP-10 (data not shown), and a higher proportion of the IFN-λ1^high^ patients belonged to the IL-17A^high^ and IL-23^high^ subgroups (Table 3).

**Table 3:** High levels of interferon-λ1 and interferon-α identify patients with different phenotypes

Characteristics	All SLE (*n* = 261)	IFN-λ1^high^ (*n* = 65)	Others (*n* = 196)	*p* Value	IFN-α^high^ (*n* = 65)	Others (*n* = 196)	*p* Value
Age, years, mean (SD)	47.6 (14)	46.3 (15)	48.0 (14)	ns	43.3 (15)	49.0 (14)	0.01
Ever smoker, %	54	40	59	0.008	57	53	ns
SLAM >6, %	49	52	48	ns	49	49	ns
SLEDAI >6, %	26	31	25	ns	29	25	ns
SDI >0, %	64.4	60	65	ns	60	65	ns
Autoantibodies, %							
Nucleosome	44	55	40	0.028	46.5	43	ns
Ro52/SSA	28	28	27	ns	41.5	22.5	0.003
Ro60/SSA	40.5	41.5	40	ns	51	37	0.05
La/SSB	21.5	20	22	ns	37	16	0.005
LA	16	20	15	ns	4.5	20	0.003
aCL IgG	19	23	18	ns	9	22.5	0.02
aβ_2_GP1 IgG	21	23	20	ns	9	25	0.009
Triple-aPL	15	18.5	13	ns	4.5	18	0.008
Mucocutaneous, %							
Mucocutaneous SLAM >0	38	38	38	ns	54	33	0.002
Reticuloendothelial, %							
Lymphadenopathy SLAM >0	13	15	12	ns	22	9.5	0.02
Musculoskeletal, %							
Arthritis	17	15	18	ns	17	17.5	ns
Musculoskeletal SDI >0	13	4.5	20.5	0.002	14	17	ns
Erosive arthritis SDI >0	10.0	1.5	13	0.009	8	11	ns
Cardiovascular, %							
Secondary APS	17	21.5	15	ns	9	20	0.058
VTE	15	17	15	ns	6	18	0.018
Any vascular event	29	31	28	ns	15	33	0.006
Renal							
Creatinine, μmol/L, mean (SD)	87 (90)	91 (124)	75 (5)	ns	70 (16)	93 (103)	0.003
GFR, ml/minute/1.73 m^2^, median (IQR)	84 (67–101)	83 (68–101)	85(67–103)	ns	91 (76–108)	82 (76–108)	0.028
Routine laboratory tests							
WBC, 10^9^/L, mean (SD)	5.2 (2.2)	4.9 (2)	5.4 (2.3)	ns	4.7 (1.9)	5.5 (2.3)	0.02
Lymphocytes, 10^9^/L, mean (SD)	1.3 (0.7)	1.08 (0.5)	1.3 (0.7)	0.003	1.2 (0.6)	1.3 (0.05)	ns
Complement							
C3, g/L, mean (SD)	0.86 (0.24	0.82 (0.26)	0.88 (0.24)	ns	0.79 (0.28)	0.89(0.2)	0.004
C4, g/L, mean (SD)	0.15 (0.07)	0.14 (0.08)	0.15 (0.07)	ns	0.12 (0.06)	0.16 (0.07)	0.001
Medication							
Warfarin, %	15	17	14	ns	5	18	0.008
Prednisone, months, mean (SD)	232 (822)	206 (390)	241 (926)	ns	88 (151)	280 (940)	0.009
Cytokines, %							
IL-17A^high^	10	34	1.5	0.0001	8	11	ns
IL-23^high^	25	55	14	0.0001	35.5	21	0.026
IP-10^high^	25	29	24	ns	30	23	ns

*Abbreviations: aβ*_*2*_ *GP1* Anti-β_2_-antiglycoprotein domain 1 antibodies, *aCL* Anticardiolipin antibodies, *aPL* Antiphospholipid antibodies, *APS* Antiphospholipid syndrome, *C3* Complement component C3, *C4* Complement component C4, *GFR* Glomerular filtration rate, *IFN* Interferon, *IgG* Immunoglobulin G, *IL* Interleukin, *IP-10* Interferon-γ-induced protein 10, *LA* Lupus anticoagulant, *ns* Nonsignificant, *SDI* Systemic Lupus International Collaborating Clinics/American College of Rheumatology Damage Index, *SLAM* Systemic Lupus Activity Measure, *SLE* Systemic lupus erythematosus, *SLEDAI* Systemic Lupus Erythematosus Disease Activity Index, *VTE* Venous thromboembolism, *WBC* White blood cells

The left column (all SLE) demonstrates distribution of investigated parameters for the whole SLE cohort. In statistical analysis, a subgroup of patients with high cytokine levels (IFN-α^high^ [*n* = 65] or IFN-λ1^high^ [*n* = 65]) was compared with all the others. Statistical analysis of continuous variables was performed by Student’s *t* test; values calculated as ratios were compared by nonparametric Wilcoxon/Kruskal-Wallis test; and proportions were compared by chi-square or Fisher’s exact test

#### Features associated with high levels of IFN-α

Patients with high IFN-α levels (*n* = 65) were compared as a group with the rest of the SLE cohort (*n* = 196). The patients in the IFN-α^high^ subgroup were younger. The antibody profile of the IFN-α^high^ group included frequent positivity for Ro52/SSA, Ro60/SSA (a trend, *p* = 0.05), and La/SSB antibodies, but the presence of LAC, aCL IgG, and aβ_2_GP1 IgG was uncommon. At the time of inclusion, a larger proportion of patients had active mucocutaneous manifestations and lymphadenopathy. A history of VEs and treatment with anticoagulants was less common in the IFN-α^high^ group. Our data suggest that these patients had better-preserved renal function, as indicated by lower creatinine levels and higher GFR. The leukocyte counts and complement levels were lower in comparison with those of the other patients (Table 3).

#### Features associated with simultaneous upregulation of IFN-α and IFN-λ1

In these analyses, patients with high levels of both IFN-α and IFN-λ1 (*n* = 19) were compared with the rest of the sample (*n* = 242). Despite the lack of correlation between IFN-λ1 and IFN-α (Fig. 1a), 14.5% of patients had detectable levels of both IFNs, and 7.3% patients had high expression of both. Double-high (IFN-α^high^ and IFN-λ1^high^) patients were younger and had shorter duration of SLE. They also tested positive more often for Ro52/SSA and Ro60/SSA antibodies (Table 4). A significant proportion of double-high patients had active disease (SLEDAI >6 and SLAM >6). The characteristic phenotype of active SLE included lymphadenopathy, cortical dysfunction, history of seizures, leukopenia and lymphopenia, and low C4 level, but better-preserved renal function, as suggested by lower creatinine. The double-high patients were treated with steroids for a shorter time (Table 4). A significant proportion of these patients also had high levels of IL-23 (Table 4).

**Table 4:** Double-high interferon-λ1 and interferon-α as well as high interferon-γ-induced protein 10 define two subgroups of active patients with distinct phenotypes

Characteristics	All SLE (*n* = 261)	IFN-α^high^ + IFN-λ1^high^ (*n* = 19)	Others (*n* = 242)	*p* value	IP-10^high^ (*n* = 63)	Others (*n* = 198)	*p* value
Age, years, mean (SD)	47.6 (14)	39.5 (13)	48 (14)	0.01	47 (14)	47.9 (15)	ns
SLE duration, years, mean (SD)	15 (11)	11 (7.7)	15 (11)	0.02	15 (10)	15 (11)	ns
SLAM, median (IQR)	6 (4–10)	9 (6–13)	6 (4–10)	0.03	8 (4–12)	6 (3.75–9)	0.02
SLAM >6, %	49	73	47	0.03	59	46	0.07
SLEDAI >6, %	26	53	24	0.01	41	20	0.0008
SDI >0, %	64	47	66	ns	65	65	ns
Autoantibodies, %							
Nucleosome	44	58	42.5	ns	60	38	0.002
Ro52/SSA	28	58	25	0.002	36.5	23.5	0.046
Ro60/SSA	40.5	68.5	38	0.01	49	38	ns
La/SSB	21.5	37	20	0.09	27	19.5	ns
Mucocutaneous, %							
Mucocutaneous SLAM >0	37	55	36	ns	40	36	ns
Reticuloendothelial, %							
Lymphadenopathy SLAM, >0	13	35	11	0.003	16	12	ns
Musculoskeletal, %							
Arthritis	17	16	17.5	ns	29	14	0.009
Musculoskeletal SDI >0	13	0	18	0.05	17.5	17	ns
Cardiovascular, %							
Pleuritis/carditis SLAM, >0	6.3	17	5.5	0.06	10	5.5	ns
Any vascular event	29	10.5	30	0.06	32	26	ns
Neurologic, %							
Cortical dysfunction SLAM >0	17	37	15	0.01	19	16.5	na
Seizures, ever	11	26	9.5	0.02	14	10	ns
Lupus headache SLAM >0	36	37	35	ns	25	40	0.03
Renal							
Creatinine, μmol/L, mean (SD)	87 (90)	68 (12)	89 (93)	0.002	91 (92)	86 (91)	ns
Routine laboratory tests							
WBC, 10^9^/L, mean (SD)	5.2 (2.2)	3.9 (1.2)	5.4 (2.2)	<0.0001	4.7 (2.2)	5.4 (2.2)	0.03
Lymphocytes, 10^9^/L, mean (SD)	1.3 (0.7)	1.03 (0.4)	1.3 (0.7)	0.04	1.07 (0.57)	1.3(0.7)	0.01
Complement components							
C3, g/L, mean (SD)	0.86 (0.24	0.7 (0.3)	0.87 (0.2)	0.08	0.79 (0.3)	0.89 (0.2)	0.0004
C4, g/L, mean (SD)	0.15 (0.07)	0.12 (0.06)	0.15 (0.07)	0.049	0.13 (0.08)	0.16 (0.07)	0.001
Medication							
Prednisone, months, mean (SD)	232 (822)	54 (61)	247 (853)	0.001	157 (384)	259 (926)	ns
Cytokines							
IL-23^high^, %	25	53	22.5	0.004	42	19	0.0003

*Abbreviations: C3* Complement component C3, *C4* Complement component C4, *IFN* Interferon, *IL* Interleukin, *IP-10* Interferon-γ-induced protein 10, *ns* Nonsignificant, *SDI* Systemic Lupus International Collaborating Clinics/American College of Rheumatology Damage Index, *SLAM* Systemic Lupus Activity Measure, *SLE* Systemic lupus erythematosus, *SLEDAI* Systemic Lupus Erythematosus Disease Activity Index, *WBC* White blood cells

The left column (all SLE) demonstrates distribution of investigated parameters for the whole SLE cohort. In statistical analysis, a subgroup of patients with high cytokine levels (double-high IFN-α + IFN-λ1 [*n* = 19] or IP-10^high^ [*n* = 63]) was compared with all the other groups. Statistical analysis of continuous variables was performed by Student’s *t* test; values calculated as ratios were compared by nonparametric Wilcoxon/Kruskal-Wallis test; and proportions were compared by chi-square or Fisher’s exact test

#### Features associated with high IP-10

A total of 63 patients had high levels of IP-10 in serum. The IP-10^high^ subgroup had more active disease with higher SLEDAI scores. Patients had more active arthritis, leukopenia, lymphopenia, and low complement components C3 and C4. Interestingly, IP-10^high^ patients tested positive for both antinucleosome and anti-Ro52/SSA antibodies more often than other patients. Fewer patients in this group experienced lupus headache. A significant proportion of these patients also had high levels of IL-23 (Table 4).

#### Features associated with simultaneous upregulation of IFN-λ1, IL-17A, and IL-23

Because levels of IFN-λ1, IL-17A, and IL-23 were intercorrelated (Table 2), we analyzed patients with high levels of all three cytokines as a group (*n* = 20) and compared them with the rest of a cohort (*n* = 241). A substantial proportion (65%) of these patients had disease damage in two or more organ domains (SDI >1), and 55% had impaired renal function. Other common features were triple-aPL positivity and a history of thrombocytopenia. Higher IP-10, but not IFN-α, levels were also characteristic (Table 5). There were no other distinct clinical or laboratory features observed (data not shown).

**Table 5:** Clinical associations with the high levels of three cytokines: interferon-λ1, interleukin-17A, and interleukin-23

Characteristics	All other patients (*n* = 241)	IFN-λ1, IL-17A, and IL-23 triple-high (*n* = 20)	*p* Value
Age, years, mean (SD)	47 (14)	52.6 (16)	ns
Disease duration, years, mean (SD)	14 (11)	20 (14)	ns
SLAM >6 (%)	49.5	45	ns
SDI >1 (%)	38	65	0.02
Renal manifestations			
Nephritis, ever, %	43	35	ns
Creatinine, μmol/L, median (IQR)	69 (60–82)	87 (66.5–100)	0.02
GFR ≤60 ml/minute/1.73 m^2^, %	14	40	0.002
Renal damage, %	29	55	0.008
Laboratory parameters			
Thrombocytopenia, ever, %	19	40	0.02
Triple-aPL, %	13	30	0.04
IP-10, pg/ml, median (IQR)	175 (116–325)	323 (250–478)	0.001
IFN-α, pg/ml, median (IQR)	30 (30–740)	30 (30–622)	ns

*Abbreviations: aPL* Antiphospholipid antibodies, *GFR* Glomerular filtration rate, *IFN* Interferon, *IL* Interleukin, *IP-10* Interferon-γ-induced protein 10, *ns* Nonsignificant, *SDI* Systemic Lupus International Collaborating Clinics/American College of Rheumatology Damage Index, *SLAM* Systemic Lupus Activity Measure

Statistical analysis of continuous variables was performed by Student’s *t* test; values calculated as ratios were compared by nonparametric Wilcoxon/Kruskal-Wallis test; and proportions were compared by chi-square or Fisher’s exact test

Analysis of treatment regimens, including steroid doses, as well as types and doses of disease-modifying antirheumatic drugs (DMARDs), including rituximab, did not identify any differences among any of the investigated groups (data not shown).

## Discussion

This is, to the best of our knowledge, the first comparative analysis of serum levels of types I and III IFNs in patients with SLE and matched population control subjects. The levels of both IFNs were higher in patients with SLE. Interestingly, increased and even high levels of IFN-λ1 and IFN-α could also be detected in a proportion of control subjects, both in patients with other diagnoses and in apparently healthy individuals. Our well-characterized SLE cohort enabled distinct phenotypic stratification of patients based on cytokine profiling. We report that both IFN types are increased in subgroups of patients with SLE, but only with a partial overlap and with no correlation. Furthermore, we demonstrate that high levels of IFN-λ1 and IFN-α are associated with different clinical and serological profiles. IFN-λ1 correlates with classical Th17 cytokines, and the subset of patients with high levels of IFN-λ1, IL-17A, and IL-23 is characterized by more organ damage, in particular renal impairment. Moreover, we identified two distinct subsets of patients with active SLE: one with high levels of both IFN-λ1 and IFN-α and another with high levels of IP-10.

Our data indicate that specific antibody patterns are associated with different cytokine profiles. We found two nonoverlapping subpopulations positive for antinucleosome antibodies: one IFN-λ1^high^ and another IP-10^high^. The latter association has been reported before [32]. Interestingly, antinucleosome antibodies have been suggested to be even more specific than anti-dsDNA and predict lupus nephritis [33, 34]. We found that the IFN-α^high^ group, in line with previous reports, was more often positive for Ro/SSA and La/SSB antibodies [8]. We report a novel observation that positivity for aPL and VEs are less common in the IFN-α^high^ group.

In SLE, type III IFNs have scarcely been studied, and this is the first large study where clinical associations could be thoroughly investigated [11]. We found that musculoskeletal involvement is uncommon among IFN-λ1^high^ patients. In an earlier, smaller Asian study, researchers reported that levels of IFN-λ1 correlate to SLEDAI and that patients with renal and/or arthritic manifestations have higher serum levels of IFN-λ1 [11]. We could not directly confirm these associations. This might be due to genetic differences but also to a different study design (ours being cross-sectional and the other one at disease exacerbation). Interestingly, other investigators have reported that IFN-λ2 had a therapeutic effect in collagen-induced arthritis model through reduction of Th17 cells [12]. In addition, we found a correlation between IFN-λ1, IL-17A, and IL-23 and that the triple-high subgroup displays higher SDI scores and more renal impairment. Moreover, we found that positivity for all three aPL antibodies and a history of thrombocytopenia are common characteristics of this subgroup. We and others have previously reported that high IL-17, IL-23, and aPL are associated with poor nephritis outcomes [18, 35, 36]. Thus, our data imply that type III IFNs, together with Th17 cytokines, rather than IFN-α, are associated with an unfavorable nephritis prognosis.

Data on types I and III IFNs and mucocutaneous lupus are somewhat conflicting [8, 16]. Increased IFN-λ1 expression in serum and skin lesions in patients with cutaneous lupus erythematosus has been reported, but our present study and previous reports indicate that the mucocutaneous inflammation in SLE is most probably driven by type I IFNs [8, 16].

We further report leukopenia in the IFN-α^high^ group and lymphopenia in the IFN-λ1^high^ group, whereas the double-IFN^high^ groups were low in both leukocytes and lymphocytes. Triple-high IFN-λ1, IL-17A, and IL-23 patients displayed a higher frequency of thrombocytopenia. Hence, different hematological manifestations seem to be associated with different cytokine patterns.

So far, data regarding the association between IFN signature and disease activity have been of a dual nature [6, 7, 37]. According to our findings, upregulation of both types I and III IFNs is associated with high disease activity and a phenotype comprising neuropsychiatric involvement, lymphadenopathy, leukopenia and lymphopenia, Ro/SSA positivity, and low C4 level. Further, in our cohort, upregulation of IP-10 was not, as often assumed, a proxy for IFN-α activity [19]. Rather, levels of IP-10 were independently associated with high disease activity. In addition, active arthritis was more common in this group. IP-10 is also regarded as a disease activity marker in rheumatoid arthritis, and our findings indicate that it might be a marker of lupus arthritis [38]. Altogether, our findings indicate that patients with active SLE, but with different phenotypes, can be identified by either high serum levels of both IFN-λ1 and IFN-α or high levels of IP-10.

The clinical profile of the IFN-α^high^ subgroup in our cohort is in line with previous studies [8]. Our novel observations are that the IFN-α^high^ group had better preserved renal function, was less often positive for aPL, and had fewer VEs. Hence, only a few of them were receiving warfarin treatment. Our results support previous observations that the risk for cardiovascular morbidity and mortality (cardiovascular disease [CVD]) is associated with positivity for aPL, whereas patients with Ro/SSA and La/SSB positivity have reduced risk [1, 39]. In the present study, we further define that the group with lower CVD risk had high IFN-α levels. Positive associations between activation of IFN-regulated genes and CVD were previously reported. However, specific IFN levels were not investigated in this context, which is of importance because type III IFNs also contribute to the IFN signature [40].

Our study included measurement of IFN-α and IFN-λ1 levels in a large cohort of population control subjects. A proportion of control subjects had detectable—and some had high—levels of the investigated cytokines. The study design included population control subjects matched with patients with SLE for age, sex, and geographical region (i.e., individuals with other autoimmune diseases, malignancies, or chronic infection [hepatitis] were included). This might partially explain a fairly high proportion of control subjects with increased cytokine levels. IFNs are part of a physiological immune response against viruses, and no screening for possible subclinical viral infections was performed at inclusion, though no control individuals with acute infections were recruited. Our findings implicate that tracking and interpreting upregulation of IFNs in humans is complicated because these cytokines are part of physiological antiviral protection. Upregulation of IFN-α and IFN-λ1 is not specific for SLE. However, we observed certain associations among SLE phenotypes and cytokine patterns. Our findings might be of interest while tailoring treatment for some SLE subsets.

Until recently, commercial ELISAs for IFN-α detection were assumed to be not sensitive enough to detect circulating IFN-α. The majority of available data on type I IFNs in SLE is therefore based on assessments of IFN-regulated gene expression scores (signatures), either in patients’ own cells or in cell lines exposed to serum from patients with SLE. The readout of these assays is upregulation of combinations of IFN-regulated genes. These methods are indirect and unspecific, and studies are therefore difficult to interpret and compare [41, 42]. Moreover, the signatures of types III and I IFNs overlap; for example, *IFIT1*, *IFI44*, *STAT1*, *CXCL-10/IP-10*, *IP-9*, and *Mx1* are all induced by both IFN types. Therefore, an impact of IFN-λ subtypes should be considered in the context of a positive IFN signature in SLE [7, 17, 43, 44]. Various impacts of IFN-λ and IFN-α subtypes could also explain contrasting clinical associations in previous reports [7, 8, 23, 45]. The pan-IFN-α and pan-IFN-λ1 ELISAs used in this study represent a novel, direct approach to measuring circulating types I and III IFNs. We observed similar clinical associations as reported in an earlier study in which investigators used a direct method to measure IFN-α [8]. This is reassuring and suggests that this method could serve as a direct, cost-effective way to measure circulating IFNs. Further evaluation is needed to confirm its reliability. To increase accuracy, we limited our comparisons to groups with substantially high IFN levels.

We analyzed the possible impact of steroids and/or DMARDs, but we could not identify any associations. A weakness of this study is the cross-sectional approach whereby the majority of patients were included in a stable phase of inactive or low-activity disease. Inclusion of patients at SLE diagnosis or during active flares is planned.

Importantly, therapeutic blockade of the IFN-α pathway by the IFN-α receptor (IFN-AR) antagonist anifrolumab is being evaluated in clinical trials as a novel therapy for SLE [21]. Investigators reported that patients with an IFN signature and rash respond better to this treatment. Our findings suggest that probably not all patients with SLE will be candidates for targeting the type I IFN pathway. The results of this study imply that patients with SLE nephritis may derive limited benefit from IFN-AR blockade but could instead be candidates for targeting either type III IFNs or the Th17 axis.

## Conclusions

We present the first comparative study on levels of circulating types I and III IFNs in a large cohort of consecutive patients with SLE and control subjects. Our results demonstrate that levels of IFN-λ1 and IFN-α do note correlate. Rather, IFN-λ1 together with associated Th17 cytokines and IFN-α characterize two distinct SLE subgroups. Triple-upregulation of IFN-λ1, IL-17A, and IL-23 associates with more disease damage, particularly renal damage. There are at least two different subgroups of patients with active disease: One is defined by simultaneously high IFN-λ1 and IFN-α and neuropsychiatric manifestations, and the second is characterized by high levels of IP-10 and lupus arthritis.

## Abbreviations

aβ_2_GP1: Anti-β_2_-antiglycoprotein domain 1 antibodies
aCL: Anticardiolipin antibodies
ANA: Antinuclear antibodies
aPL: Antiphospholipid antibodies
APS: Antiphospholipid syndrome
C3: Complement component C3
C4: Complement component C4
CVD: Cardiovascular disease
CXCL10: C-X-C motif chemokine 10
DMARD: Disease-modifying antirheumatic drug
dsDNA: Double-stranded DNA
ELISA: Enzyme-linked immunosorbent assay
GFR: Glomerular filtration rate
IFN: Interferon
IFN-AR: Interferon-α receptor
IgG: Immunoglobulin G
IL: Interleukin
IP-10: Interferon-γ-induced protein 10
LA: Lupus anticoagulant
nd: Not done
ns: Nonsignificant
RT: Room temperature
SDI: Systemic Lupus International Collaborating Clinics/American College of Rheumatology Damage Index
SLAM: Systemic Lupus Activity Measure
SLE: Systemic lupus erythematosus
SLEDAI: Systemic Lupus Erythematosus Disease Activity Index
Th17: T-helper type 17 cells
VE: Vascular event
VTE: Venous thromboembolism
WBC: White blood cells

## References

[1] Gustafsson JT, Simard JF, Gunnarsson I, Elvin K, Lundberg IE, Hansson LO (2012). Risk factors for cardiovascular mortality in patients with systemic lupus erythematosus, a prospective cohort study. Arthritis Res Ther..

[2] Vikerfors A, Johansson AB, Gustafsson JT, Jönsen A, Leonard D, Zickert A (2013). Clinical manifestations and anti-phospholipid antibodies in 712 patients with systemic lupus erythematosus: evaluation of two diagnostic assays. Rheumatology (Oxford).

[3] Kvarnström M, Dzikaite-Ottosson V, Ottosson L, Gustafsson JT, Gunnarsson I, Svenungsson E (2013). Autoantibodies to the functionally active RING-domain of Ro52/SSA are associated with disease activity in patients with lupus. Lupus..

[4] Rönnblom LE, Alm GV, Oberg KE (1991). Autoimmunity after alpha-interferon therapy for malignant carcinoid tumors. Ann Intern Med..

[5] Munroe ME, Lu R, Zhao YD, Fife DA, Robertson JM, Guthridge JM (2016). Altered type II interferon precedes autoantibody accrual and elevated type I interferon activity prior to systemic lupus erythematosus classification. Ann Rheum Dis..

[6] Rönnblom L, Eloranta ML (2013). The interferon signature in autoimmune diseases. Curr Opin Rheumatol..

[7] Kirou KA, Lee C, George S, Louca K, Peterson MGE, Crow MK (2005). Activation of the interferon-α pathway identifies a subgroup of systemic lupus erythematosus patients with distinct serologic features and active disease. Arthritis Rheum..

[8] Bengtsson AA, Sturfelt G, Truedsson L, Blomberg J, Alm G, Vallin H (2000). Activation of type I interferon system in systemic lupus erythematosus correlates with disease activity but not with antiretroviral antibodies. Lupus..

[9] Kirou KA, Lee C, George S, Louca K, Papagiannis IG, Peterson MGE (2004). Coordinate overexpression of interferon-α-induced genes in systemic lupus erythematosus. Arthritis Rheum..

[10] Rusinova I, Forster S, Yu S, Kannan A, Masse M, Cumming H (2013). Interferome v2.0: an updated database of annotated interferon-regulated genes. Nucleic Acids Res.

[11] Wu Q, Yang Q, Lourenco E, Sun H, Zhang Y (2011). Interferon-λ1 induces peripheral blood mononuclear cell-derived chemokines secretion in patients with systemic lupus erythematosus: its correlation with disease activity. Arthritis Res Ther..

[12] Blazek K, Eames HL, Weiss M, Byrne AJ, Perocheau D, Pease JE (2015). IFN-λ resolves inflammation via suppression of neutrophil infiltration and IL-1β production. J Exp Med..

[13] Sheppard P, Kindsvogel W, Xu W, Henderson K, Schlutsmeyer S, Whitmore TE (2003). IL-28, IL-29 and their class II cytokine receptor IL-28R. Nat Immunol..

[14] Yin Z, Dai J, Deng J, Sheikh F, Natalia M, Shih T (2012). Type III IFNs are produced by and stimulate human plasmacytoid dendritic cells. J Immunol..

[15] Wolk K, Witte K, Witte E, Raftery M, Kokolakis G, Philipp S (2013). IL-29 is produced by T_H_17 cells and mediates the cutaneous antiviral competence in psoriasis. Sci Transl Med.

[16] Zahn S, Rehkämper C, Kümmerer BM, Ferring-Schmidt S, Bieber T, Tüting T (2011). Evidence for a pathophysiological role of keratinocyte-derived type III interferon (IFNλ) in cutaneous lupus erythematosus. J Invest Dermatol..

[17] Mordstein M, Neugebauer E, Ditt V, Jessen B, Rieger T, Falcone V (2010). Lambda interferon renders epithelial cells of the respiratory and gastrointestinal tracts resistant to viral infections. J Virol..

[18] Zickert A, Amoudruz P, Sundström Y, Rönnelid J, Malmström V, Gunnarsson I (2015). IL-17 and IL-23 in lupus nephritis - association to histopathology and response to treatment. BMC Immunol..

[19] Rose T, Grützkau A, Hirseland H, Huscher D, Dähnrich C, Dzionek A (2013). IFNα and its response proteins, IP-10 and SIGLEC-1, are biomarkers of disease activity in systemic lupus erythematosus. Ann Rheum Dis..

[20] Bauer JW, Petri M, Batliwalla FM, Koeuth T, Wilson J, Slattery C (2009). Interferon-regulated chemokines as biomarkers of systemic lupus erythematosus disease activity: a validation study. Arthritis Rheum..

[21] Merrill JT, Furie R, Werth VP, Khamashta M, Drappa J, Wang L (2016). Anifrolumab reduces disease activity in multiple organ domains in moderate to severe systemic lupus erythematosus (SLE) [abstract THU0295]. Ann Rheum Dis.

[22] Tan EM, Cohen AS, Fries JF, Masi AT, McShane DJ, Rothfield NF (1982). The 1982 revised criteria for the classification of systemic lupus erythematosus. Arthritis Rheum..

[23] Castrejón I, Tani C, Jolly M, Huang A, Mosca M (2014). Indices to assess patients with systemic lupus erythematosus in clinical trials, long-term observational studies, and clinical care. Clin Exp Rheumatol.

[24] Liang MH, Socher SA, Roberts WN, Esdail JM (1988). Measurement of systemic lupus erythematosus activity in clinical research. Arthritis Rheum..

[25] Bombardier C, Gladman DD, Urowitz MB, Caron D, Chang CH, Committee on Prognosis Studies in SLE (1992). Derivation of the SLEDAI: a disease activity index for lupus patients. Arthritis Rheum.

[26] Urowitz MB, Gladman DD (1998). Measures of disease activity and damage in SLE. Baillieres Clin Rheumatol..

[27] Strand V, Gladman D, Isenberg D, Petri M, Smolen J, Tugwell P (1999). Outcome measures to be used in clinical trials in systemic lupus erythematosus. J Rheumatol..

[28] Klahr S (1989). The modification of diet in renal disease study. N Engl J Med..

[29] Stoll T, Seifert B, Isenberg DA (1996). SLICC/ACR Damage Index is valid, and renal and pulmonary organ scores are predictors of severe outcome in patients with systemic lupus erythematosus. Br J Rheumatol..

[30] Gustafsson JT, Gunnarsson I, Källberg H, Pettersson S, Zickert A, Vikerfors A (2015). Cigarette smoking, antiphospholipid antibodies and vascular events in systemic lupus erythematosus. Ann Rheum Dis..

[31] Miyakis S, Lockshin MD, Atsumi T, Branch DW, Brey RL, Cervera R (2006). International consensus statement on an update of the classification criteria for definite antiphospholipid syndrome (APS). J Thromb Haemost..

[32] Yin Z, Huang J, He W, Cao Z, Luo X, Zhang C (2014). Serum level of eight cytokines in Han Chinese patients with systemic lupus erythematosus using multiplex fluorescent microsphere method. Cent Eur J Immunol..

[33] Manson JJ, Ma A, Rogers P, Mason LJ, Berden JH, van der Vlag J (2009). Relationship between anti-dsDNA, anti-nucleosome and anti-alpha-actinin antibodies and markers of renal disease in patients with lupus nephritis: a prospective longitudinal study. Arthritis Res Ther..

[34] Bizzaro N, Villalta D, Giavarina D, Tozzoli R (2012). Are anti-nucleosome antibodies a better diagnostic marker than anti-dsDNA antibodies for systemic lupus erythematosus? A systematic review and a study of metanalysis. Autoimmun Rev..

[35] Koga T, Ichinose K, Tsokos GC. T cells and IL-17 in lupus nephritis. Clin Immunol. doi:10.1016/j.clim.2016.04.010. In press. 10.1016/j.clim.2016.04.010 PMC5074925 27109641

[36] Gerhardsson J, Sundelin B, Zickert A, Padyukov L, Svenungsson E, Gunnarsson I (2015). Histological antiphospholipid-associated nephropathy versus lupus nephritis in patients with systemic lupus erythematosus: an observational cross-sectional study with longitudinal follow-up. Arthritis Res Ther..

[37] Jolly M, Francis S, Aggarwal R, Mikolaitis RA, Niewold TB, Chubinskaya S (2014). Serum free light chains, interferon-alpha, and interleukins in systemic lupus erythematosus. Lupus..

[38] Kuan WP, Tam LS, Wong CK, Ko FWS, Li T, Zhu T (2010). CXCL 9 and CXCL 10 as sensitive markers of disease activity in patients with rheumatoid arthritis. J Rheumatol..

[39] Gustafsson J, Gunnarsson I, Börjesson O, Pettersson S, Möller S, Fei GZ (2009). Predictors of the first cardiovascular event in patients with systemic lupus erythematosus - a prospective cohort study. Arthritis Res Ther..

[40] Somers EC, Zhao W, Lewis EE, Wang L, Wing JJ, Sundaram B (2012). Type I interferons are associated with subclinical markers of cardiovascular disease in a cohort of systemic lupus erythematosus patients. PLoS One..

[41] Niewold TB, Hua J, Lehman TJA, Harley JB, Crow MK (2007). High serum IFN-α activity is a heritable risk factor for systemic lupus erythematosus. Genes Immun..

[42] Lood C, Blanco LP, Purmalek MM, Carmona-Rivera C, De Ravin SS, Smith CK (2016). Neutrophil extracellular traps enriched in oxidized mitochondrial DNA are interferogenic and contribute to lupus-like disease. Nat Med..

[43] Mordstein M, Michiels T, Staeheli P (2010). What have we learned from the IL28 receptor knockout mouse?. J Interferon Cytokine Res..

[44] Dickensheets H, Sheikh F, Park O, Gao B, Donnelly RP (2013). Interferon-lambda (IFN-λ) induces signal transduction and gene expression in human hepatocytes, but not in lymphocytes or monocytes. J Leukoc Biol..

[45] Feng X, Wu H, Grossman JM, Hanvivadhanakul P, FitzGerald JD, Park GS (2006). Association of increased interferon-inducible gene expression with disease activity and lupus nephritis in patients with systemic lupus erythematosus. Arthritis Rheum..

